# Demonstration of high-speed and low-complexity continuous variable quantum key distribution system with local local oscillator

**DOI:** 10.1038/s41598-021-88468-1

**Published:** 2021-05-04

**Authors:** Shengjun Ren, Shuai Yang, Adrian Wonfor, Ian White, Richard Penty

**Affiliations:** 1 Centre for Photonic Systems, University of Cambridge, Cambridge, CB3 0FA UK; 2 University of Bath, Claverton Down, Bath, BA2 7AY UK

**Keywords:** Quantum information, Optical physics, Electrical and electronic engineering

## Abstract

We present an experimental demonstration of the feasibility of the first 20 + Mb/s Gaussian modulated coherent state continuous variable quantum key distribution system with a locally generated local oscillator at the receiver (LLO-CVQKD). To increase the signal repetition rate, and hence the potential secure key rate, we equip our system with high-performance, wideband devices and design the components to support high repetition rate operation. We have successfully trialed the signal repetition rate as high as 500 MHz. To reduce the system complexity and correct for any phase shift during transmission, reference pulses are interleaved with quantum signals at Alice. Customized monitoring software has been developed, allowing all parameters to be controlled in real-time without any physical setup modification. We introduce a system-level noise model analysis at high bandwidth and propose a new ‘combined-optimization’ technique to optimize system parameters simultaneously to high precision. We use the measured excess noise, to predict that the system is capable of realizing a record 26.9 Mb/s key generation in the asymptotic regime over a 15 km signal mode fibre. We further demonstrate the potential for an even faster implementation.

## Introduction

Quantum key distribution (QKD) allows a common random secure key exchange between two authenticated users, Alice and Bob, connected via an insecure quantum channel that can be manipulated by an eavesdropper—Eve^1–4^. Unlike conventional public-key cryptography which assumes that the required computational complexity is too great for practical breaking of encryption, the critical information in a QKD system is unconditionally protected by the fundamental laws of quantum mechanics^5–7^. In contrast to dedicated single-photon detector based discrete variable (DV) QKD systems, continuous variable QKD (CVQKD) modulates both quadratures of the electromagnetic field with continuous random data which can be later extracted with shot noise limited coherent detection techniques^8,9^ and post-processed to distil secure keys. More importantly, CVQKD systems have the benefits of compatibility with commercial off-the-shelf (COTS) telecom components and exhibit high detection efficiency, enabling low-cost^10^ and possibly higher secret key rates over access and metro Dense Wavelength Division Multiplexing (DWDM) network distances^11–13^. The Gaussian-modulated coherent-state (GMCS) protocol of CVQKD has undergone rigorous development to offer both theoretical and experimental security against malicious eavesdropping attacks^14–16^. However, several obstacles have been identified in practical CVQKD systems, especially those associated with a co-transmitted local oscillator (LO) between Alice and Bob. First, the LO can be accessed by Eve, which could result in security loopholes that compromise the secure key generation. Several attacks have been identified which manipulate the LO, for example, LO intensity fluctuation attack^17^, calibration attack^18^ and wavelength attack^19^. Second, owing to the attenuation loss in the quantum channel, the attenuated LO power arriving at Bob fails to support shot-noise-limited detection over long distances^20^. Third, to achieve a high-efficiency quadrature detection at Bob, the necessary LO intensity required is typically eight orders of magnitude higher than that of the quantum signal. Such a large power disparity requires a dedicated separation technique to mitigate the photon scatter contamination from LO to quantum signals^21,22^.

To overcome these limitations, a second independent narrow linewidth laser of the same centre wavelength located at Bob has been proposed^20–22^ to realize a fully protected local LO (LLO). In order to phase lock the independent lasers at two remote ends and ensure the secure key distillation, Alice shares low-intensity reference pulses with Bob to recover the phase drift during transmission and uses a phase rotation scheme to correct for the quadrature measurements in the later reconciliation stage^23,24^. With the growing understanding of the LLO CVQKD noise models, various LLO protocols^25–32^ have been proposed to enhance different aspects of the system performance at the cost of increased system complexity. The primary goal of practical QKD development has been to increase the secret key rate with cost-effective and low complexity system configurations. However, the repetition rates of the GMCS LLO-CVQKD system demonstrations to date have been less than 100 MHz^22,29,30^, which limit the asymptotic secret key rate to the 7 Mb/s level at metropolitan network scales.

In this work, we propose and demonstrate experimentally a 500 MHz, low-complexity LLO-CVQKD system based on the GMCS protocol. A record asymptotic key rate of 26.9 Mb/s over a 15 km optical fibre length is predicted from experimental results. Higher repetition rates, and hence secure key rates, are expected with higher bandwidth signal generators. In our setup, several AC coupled wideband drivers, and high bandwidth balanced homodyne detectors are employed to break the bandwidth limitation of DC coupled components and low-speed detectors^33^. Since the previous sequential-LLO CVQKD demonstrations have been run at low repetition rates and the sequentially generated quantum signal and reference signal pulses have relatively large time intervals, several approaches with increased system complexity have been deployed to minimize the phase drift noise induced from the large pulse intervals^23,26,28^. In contrast, in our system, the high repetition rate leads to very short pulse intervals, resulting in low phase drift noise, enabling us to achieve better performance with a lower complexity configuration.

The customized software enables all system parameters to be easily adjusted without any setup modification. Based upon the noise model analysis at a high repetition rate in this paper, we propose a new ‘combined-optimization’ technique, where two of the most influential parameters for excess noise, modulation variance and reference pulse intensity, are jointly optimized to enhance the predicted key rate performance beyond 20 Mb/s. By implementing the real-time shot noise calibration, the photon contamination resulting from scattering from adjacent reference pulses is mitigated to maintain a stable low excess noise. The experimental excess noise results over the SMF link are used to validate the combined-optimization method and prove the feasibility of 20 Mb/s-level key generation over metropolitan area distances.

## Results

### System experimental design

**Figure 1:**
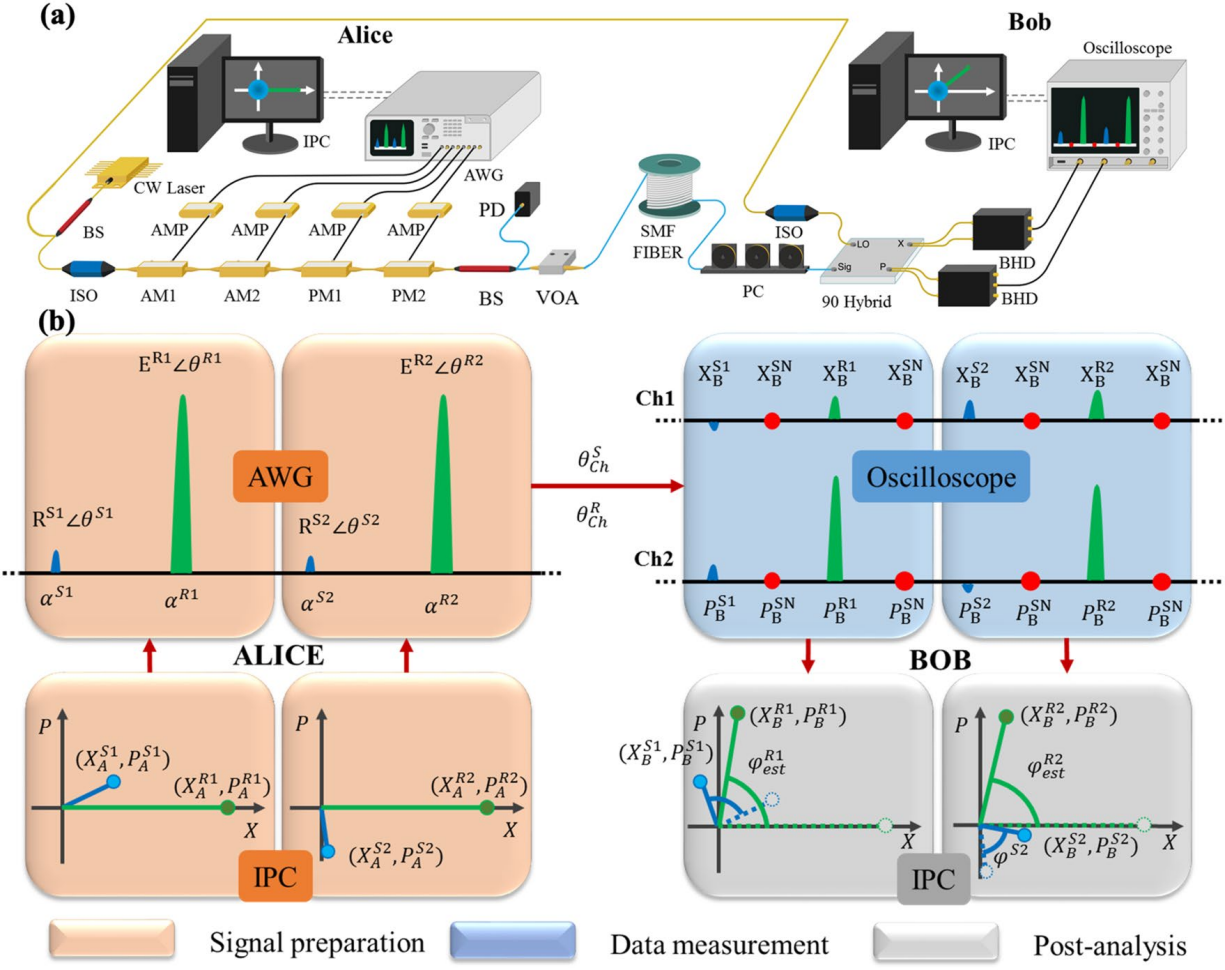
Experiment setup and data processing unit of our LLO CVQKD system. (**a**). Overall system configuration. The system has been tested with a second laser at Bob and the experimental results are equivalent to those presented here. The SMF length (15 km) is significantly longer than the coherence length of the laser (954 m). CW: continuous wave, AWG: arbitrary wave generator, BS: beam splitter, ISO: isolator, AMP: AC coupled wideband amplifier, AM: amplitude modulator, PM: phase modulator, VOA: variable optical attenuator, PD: photodiode; SMF: single mode fibre, PC: polarization controller, 90 Hybrid: 90 degree optical hybrid, BHD: balanced homodyne detector. (**b**). Schematic representation of the data processing unit along with the phase recovery scheme. In Alice’s signal preparation stage (orange region), quadratures of quantum signal pulses (blue) and reference pulses (green) are generated in an industrial PC (IPC) and converted into phase-space representation for signal modulation in the AWG. At Bob, the data measurement process (blue region) collects the X and P of signal pulses, reference pulses and real-time shot noise (red) from Ch1 and Ch2 of the oscilloscope. Then, the post-analysis (grey region) is implemented to recover the phase rotation ${\mathrm{\varphi }}^{\mathrm{S}}$ (blue angle) by estimating the rotation in the reference phase ${\mathrm{\varphi }}_{\mathrm{est}}^{\mathrm{R}}$ (green angle).

The experimental setup of the high-speed GMCS-LLO-CVQKD system using COTS optical communication components is shown in Fig. 1a. For a commercially installed system, it is clear that two separated laser sources with the same wavelength will be used at Alice and Bob. We emphasize that we have done the experiments with two independent lasers by manually tuning their wavelengths. When the two lasers’ wavelengths are matched, the experimental results and noise performance are equivalent to those collected by laser splitting configuration presented in Fig. 1a. After verifying this, a single frequency laser (SFL, Thorlabs) at 1549.73 nm with a linewidth of about 100 kHz is used, for the convenience of demonstration, as both the laser source at Alice and the local oscillator (LO) at Bob. Since the coherence length of the source is far less than that of the optical link, the quantum and reference signals arriving at Bob after a long fibre link and the LO signal arriving at Bob after the 3 m beam splitter will be incoherent. A 10:90 beam splitter (BS, Thorlabs) is used to split the laser output, and an optical isolator is located at each arm to avoid back reflections. At Alice, the continuous wave (CW) light is first carved into 0.2 ns pulses at a rate ${f}_{pulse}$ of 1 GHz using a 10 GHz LiNbO_3_ intensity modulator (AM1, iXblue). Then, the pulse amplitude is modulated by a second 10 GHz LiNbO_3_ intensity modulator (AM2, iXblue) with a high extinction ratio, and the phase is modulated by a cascaded pair of 10 GHz electro-optic phase modulators (PM1, PM2, iXblue). The pulse train is converted into interleaved quantum signals and reference pulses, one delayed by 1 ns relative to the other. All synchronization and modulation signals are generated by an arbitrary-waveform generator (AWG, Tektronix AWG 5204) with four 16-bit, 5 GS/s DAC channels. Several identical, 10 GHz AC coupled wideband RF amplifiers (AMP, iXblue) are deployed to drive the modulators in the LLO CVQKD system. The DAC channels and amplifiers have been carefully characterized to ensure their linearity in terms of the signal generation and modulation. The amplified signals are then shifted to the appropriate voltage levels by the bias-tees. Although the gain of the wideband amplifiers is around 30 dB, their 1 dB compression point below 1 GHz (6.32 Vpp max output) is insufficient to provide a necessary, 0—2 $\pi$ phase modulation voltage swing (typically 8 Vpp to 12 Vpp) required for the GMCS protocol. Therefore, we use a cascaded PM configuration where two PMs (PM1 and PM2) are connected in series. Each PM is driven by an identical wideband RF amplifier and is only responsible for up to $\pi$ phase modulation. Overall the cascaded PMs allow a linear phase modulation from 0 to 2 $\pi$. The amplitude and phase of the signal pulses are randomly modulated, using a two-dimensional zero-centred Gaussian distribution with an adjustable modulation variance ${V}_{A}{N}_{o}$ (tuned by a variable optical attenuator (VOA)), where ${N}_{o}$ is the shot noise variance. In contrast, the reference pulses are modulated with a fixed intensity ${|{E}^{R}|}^{2}{N}_{o}$ and a constant zero phase. This enables the phase sharing scheme to be used to correct for fast phase drift by tracking phase changes in the reference pulses. A 10:90 BS is used to monitor the Alice modulation and a variable optical attenuator (VOA) is placed at the output of Alice to optimize the ${V}_{A}$. After that, the signal and reference pulses propagate through a 15 km standard single mode fibre before arriving at Bob.

At Bob, since the electronic noise increases with detection bandwidth^27^, it is necessary to have sufficient LO power to satisfy the shot-noise-limited detection when using the 1.6 GHz balanced homodyne detectors (BHD, Thorlabs). A 13 dBm continuous wave LO is deployed to provide a shot noise to electronic noise ratio of 10 dB at full detector bandwidth. A polarization controller (PC) is used to compensate for polarization drift from environmental perturbations during transmission. Since low-complexity single heterodyne detection is applied to measure the X and P quadratures, both quantum signals and reference pulses are fed into a 90-degree optical hybrid (Kylia) before being detected by two BHDs. A 10-bit oscilloscope (Keysight, DSOS254A) with a 2.5 GHz bandwidth and a full sampling rate of 20 GS/s is used to collect the quadrature information, and the results are sent to a computer for the $\sqrt{{\mathrm{N}}_{\mathrm{o}}}$ normalization and the following data analysis. The real-time shot noise is determined by measuring the variance between the signal and reference pulses. Consequently, the effect of the photon leakage from adjacent reference pulses can be tracked and corrected correspondingly.

It is worth noting that our experimental setup is significantly simplified compared to other multiplexing techniques, such as the delay-line setup^23^ and extra homodyne detection module for quantum signal detection^29^ and also has advantages of flexibility in parameter adjustment and high repetition rates.

### Data processing unit

The software-based data processing unit of our system consists of three parts: signal preparation at Alice, data measurement and post-analysis at Bob. The diagram of the overall data processing unit, along with a phase recovery scheme is illustrated in Fig. 1b. At Alice, to generate the coherent quantum signal state ${\mathrm{\alpha }}^{\mathrm{S}}$, a sequence of quadrature values ${\mathrm{X}}_{\mathrm{A}}^{\mathrm{S}}$ and ${\mathrm{P}}_{\mathrm{A}}^{\mathrm{S}}$ with Gaussian variance ${\mathrm{V}}_{\mathrm{A}}$ and zero mean are prepared using an industrial personal computer (IPC). Then, the Gaussian distributed quadrature values are converted into phasor form ${R}^{S}\angle {\theta }^{S}$ where ${\mathrm{R}}^{\mathrm{S}}$ is the Rayleigh distributed signal amplitude and ${\uptheta }^{\mathrm{S}}$ is the uniformly distributed signal phase. These two variables are sent to the AWG to generate the modulation signals, which are then amplified and fed into AM2 and cascaded PMs. In order to recover arbitrary phase rotation of the states in phase space, the other coherent state—reference pulse ${\mathrm{\alpha }}^{\mathrm{R}}$ of fixed publicly-announced quadrature ${\mathrm{X}}_{\mathrm{A}}^{\mathrm{R}}$ and ${\mathrm{P}}_{\mathrm{A}}^{\mathrm{R}}$ is transmitted along with each quantum signal state. For ease of phase recovery implementation, the initial reference pulse is prepared with zero-phase angle ${\theta }^{R}=0$ and fixed intensity ${|{E}^{R}|}^{2}={{X}_{A}^{R}}^{2}+{{P}_{A}^{R}}^{2}$, which is a few orders of magnitude higher than the signal variance ${V}_{A}$ but much weaker than the LO. It is of great importance that the reference pulse amplitude should not be too large, to limit the signal-reference pulse interference. Since the system repetition rate ${\mathrm{f}}_{\mathrm{rep}}$ is 500 MHz and the signal and reference pulses are alternatively produced from the light pulses generated by AM1, ${\mathrm{f}}_{\mathrm{pulse}}$ is set to 1 GHz.

At Bob, the quantum signals and reference pulses are detected with a strong LO using heterodyne measurement with a detector efficiency $\upeta$. The X and P quadrature results are collected by channel 1 and channel 2 of the 20 GS/s oscilloscope (shown in the blue region) respectively. With a 500 MHz repetition rate, every pair of quantum signal and reference pulses are oversampled by 40 sample points in every repetition period ${\mathrm{T}}_{\mathrm{rep}}$. Within each ${\mathrm{T}}_{\mathrm{rep}}$ interval, four samples from each channel are recorded and sent to the IPC for phase recovery and post-analysis, including the reference pulses’ peak values (${\mathrm{X}}_{\mathrm{B}}^{\mathrm{R}},{P}_{B}^{R})$, quantum signals’ peak values (${\mathrm{X}}_{\mathrm{B}}^{\mathrm{S}},{P}_{B}^{S})$ and real-time shot noise measurement (${\mathrm{X}}_{\mathrm{B}}^{\mathrm{SN}},{P}_{B}^{SN})$ using the middle point between each quantum signal and its adjacent reference pulse. It is worth noting that conventionally the pre-calibrated shot noise value is evaluated when only the LO is present (i.e. no signal transmission occurs). In addition, the calibrated shot noise is considered to be constant during communication. However, in practice, any fluctuations in the LO power and the photon leakage from the reference pulse’s tail will inevitably lead to some fluctuations in the real-time shot noise variance. In this paper, the signal and reference pulse quadrature values are analyzed with real-time shot noise measurement values to enhance the measurement accuracy and reduce the phase noise. Finally, as shown in the grey region in Fig. 1b, the collected data are post-analyzed to recover the phase misalignment of the two laser sources. The overall phase drift of the quantum signal ${\mathrm{\varphi }}^{\mathrm{S}}$, i.e. the phase rotation between the prepared quantum signal at Alice ${\uptheta }_{\mathrm{A}}^{S}$, and the received quantum signal at Bob ${\uptheta }_{\mathrm{B}}^{\mathrm{S}}$, can be recovered through its adjacent reference pulse by calculating the phase change ${\varphi }^{R}={\theta }_{B}^{R}-{\theta }_{A}^{R}$ along with propagation. However, due to the quantum uncertainty of the reference pulses and some practical measurement imperfections, a small amount of phase error $\phi$ exists in the phase estimation, resulting in extra phase noise in the system. The resultant phase change estimation ${\varphi }_{est}^{R}$ obtained in the experiment has $\phi$ deviation from the real quantum signal phase drift as

1 $${\varphi }^{S} = \varphi_{est}^{R} + \phi$$

With the publicly announced mean reference pulses’ quadrature values at Alice, ${{\varphi }}_{{{\text{est}}}}^{{\text{R}}}$ can be calculated based on (${\text{X}}_{{\text{B}}}^{{\text{R}}} ,P_{B}^{R} )$, as

2 $${\text{X}}_{{\text{B}}}^{{\text{R}}} = \sqrt {\frac{T\eta }{2}} \left( {X_{R}^{A} \cos {\varphi }_{{{\text{est}}}}^{{\text{R}}} + {\text{P}}_{{\text{A}}}^{{\text{R}}} \sin {\varphi }_{{{\text{est}}}}^{{\text{R}}} } \right)$$

3 $${\text{P}}_{{\text{B}}}^{{\text{R}}} = \sqrt {\frac{T\eta }{2}} \left( { - X_{R}^{A} \sin {\varphi }_{{{\text{est}}}}^{{\text{R}}} + {\text{P}}_{{\text{A}}}^{{\text{R}}} \cos {\varphi }_{{{\text{est}}}}^{{\text{R}}} } \right)$$

Without loss of generality by our prepared zero initial phase angle (i.e., ${\text{P}}_{{\text{A}}}^{{\text{R}}}$ = 0),

4 $${\varphi }_{{{\text{est}}}}^{{\text{R}}} = \tan^{ - 1} \left( {\frac{{P_{B}^{R} }}{{X_{B}^{R} }}} \right)$$

Such a phase recovery scheme has been verified to maintain the CVQKD security^21^ since the phase of the reference signal is assumed to be manipulable by Eve in standard CVQKD protocols. Consequently, the estimated phase angles are sent back to Alice for correcting the initial quadratures by

5 $$\left( {\begin{array}{*{20}c} {X_{A}^{{S^{{\prime }} }} } \\ {P_{A}^{{S^{{\prime }} }} } \\ \end{array} } \right) = \left( {\begin{array}{*{20}c} {\cos ( - {\varphi }_{{{\text{est}}}}^{{\text{R}}} )\;\;\; \sin ( - {\varphi }_{{{\text{est}}}}^{{\text{R}}} ) } \\ { - \sin ( - {\varphi }_{{{\text{est}}}}^{{\text{R}}} )\;\;\; \cos ( - {\varphi }_{{{\text{est}}}}^{{\text{R}}} )} \\ \end{array} } \right)\left( {\begin{array}{*{20}c} {X_{A}^{S} } \\ {P_{A}^{S} } \\ \end{array} } \right)$$

After the phase recovery process, the measurement bases are almost aligned between Alice and Bob. The data block size is chosen as $10^{7}$ in our experiment, and we sacrifice 10% of data for parameter estimation.

In our system, we have created a real-time data monitoring and parameter estimation application programming interface (API) to execute real-time data analysis of transmittance ${\text{ T}}$, Alice’s variance ${\text{ V}}_{{\text{A}}}$, and the excess noise ${\upxi }_{{\text{e }}}$ of each data block. The asymptotic key rate is calculated through experimental results.

### Combined-optimization algorithm

We encourage readers to read the “Methods” section before reading the following section. In the “Methods” section, we present the details of the noise model analysis of our system at high repetition rates and the corresponding secret key rate evaluation. All parameters used in our simulation and experiment are listed in Table 1 and all excess noise sources and their values are listed in Table 2. We use the notations and values defined in the “Methods” section.

**Figure 2:**
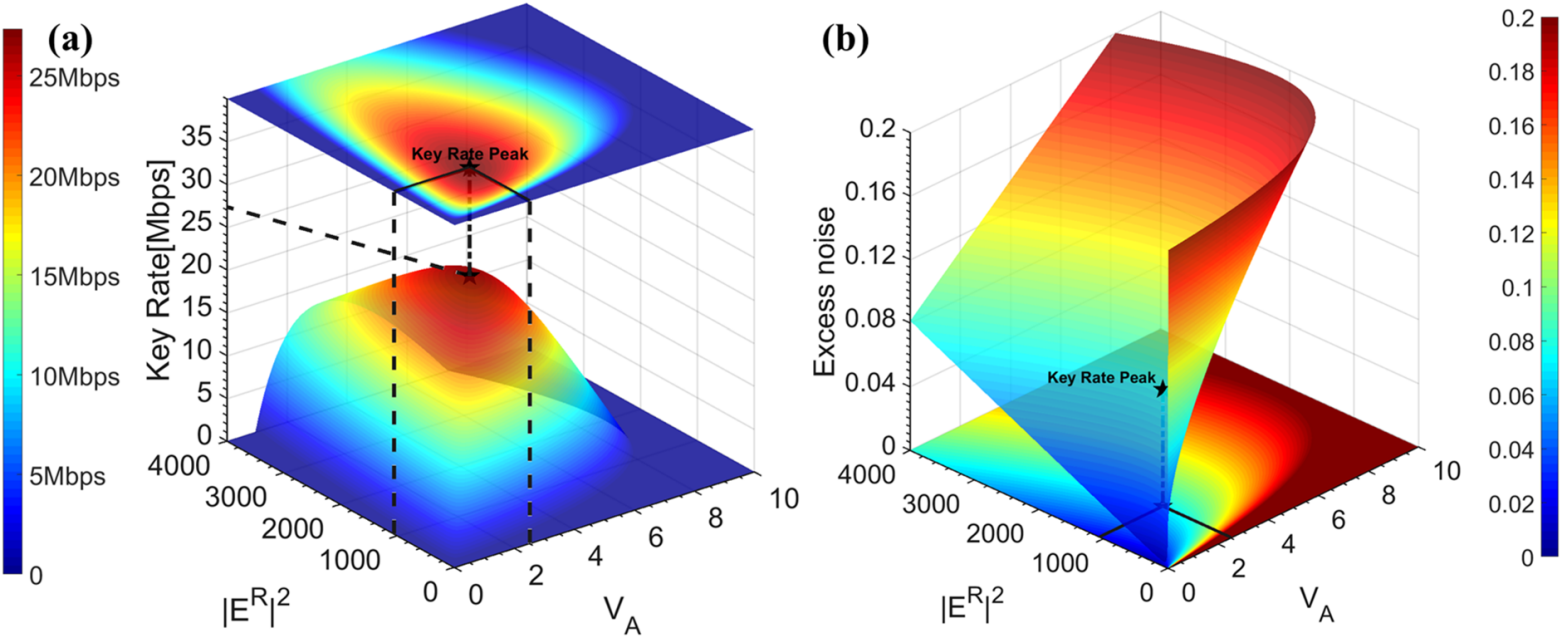
The demonstration of the proposed combined-optimization method with respect to different ${V}_{A}$= [0 10] and ${E}_{Ref}^{2}$= [0 4000] at L = 15 km and ${f}_{ref}$=500 MHz. All other parameters used in the estimations are listed in Methods section. (**a**) The secure key rate analysis for various ${V}_{A}$ and ${\left|{\mathrm{E}}^{\mathrm{R}}\right|}^{2}$. The key rate peak (the black star) in the simulation is 27.3 Mbps obtained when ${V}_{A}$=2.52 and ${\left|{\mathrm{E}}^{\mathrm{R}}\right|}^{2}$=1056. (**b**) The excess noise evaluation for various ${V}_{A}$ and ${\left|{\mathrm{E}}^{\mathrm{R}}\right|}^{2}$. The excess noise at the key rate is 0.083 and the optimal ${\left|{\mathrm{E}}^{\mathrm{R}}\right|}^{2}$ is located in the valley of the graph.

Both ${\mathrm{V}}_{\mathrm{A}}$ and ${\left|{\mathrm{E}}^{\mathrm{R}}\right|}^{2}$ play significant roles in determining the system excess noise and secure key rate. Specifically, a too weak ${\left|{\mathrm{E}}^{\mathrm{R}}\right|}^{2}$ will introduce an unacceptably large phase noise; while a large ${\left|{\mathrm{E}}^{\mathrm{R}}\right|}^{2}$ will indeed lead to less phase noise due to the more precise phase estimation. However, this will be at the expense of photon interference and other noise sources (e.g. ${\xi }_{AM}$ and ${\xi }_{ADC}$) will increase accordingly at large ${\left|{\mathrm{E}}^{\mathrm{R}}\right|}^{2}$. The choice of ${\mathrm{V}}_{\mathrm{A}}$, not only jointly determines the excess noise with the reference pulse intensity ${\left|{\mathrm{E}}^{\mathrm{R}}\right|}^{2}$, but also has a critical influence on the secret key functions in our LLO CVQKD protocol. In previous studies, the two parameters have been calibrated individually with low precision: ${\mathrm{V}}_{\mathrm{A}}$ is numerically optimized at a target distance with a maintained ${\left|{\mathrm{E}}^{\mathrm{R}}\right|}^{2}$ ^29^, while the number of optimal reference pulse photons or ${\left|{\mathrm{E}}^{\mathrm{R}}\right|}^{2}/{V}_{A}$ is normally selected in the regime where ${\mathrm{V}}_{\mathrm{A}}$ is fixed^28,34^. However, to achieve an optimized system performance, it is necessary to evaluate these two interrelated parameters conjointly. We propose for the first time a combined-optimization method to simultaneously calibrate the two parameters. The combined-optimization method uses a 3-dimensional value optimization where the system performance (secure key rate or excess noise) is evaluated by ${V}_{A}$ ranging from 0 to 10 with a resolution of 0.01, and the reference pulse intensity ${\left|{\mathrm{E}}^{\mathrm{R}}\right|}^{2}$ varies between 0 and 4000 with an interval of 1. All values are normalized to shot noise units. Following the analysis of our system shown above, the theoretical system performances at 15 km, in terms of secure key rate and excess noise, for various ${V}_{A}$ and ${\left|{\mathrm{E}}^{\mathrm{R}}\right|}^{2}$ are shown in Fig. 2a,b, respectively.

The combined-optimization results among ${V}_{A}$, ${\left|{\mathrm{E}}^{\mathrm{R}}\right|}^{2}$ and key rate are shown in Fig. 2a. The secret key rate relies heavily on both parameters, and the global maximum can be estimated using our combined searching method. The peak key rate of 27.3 Mb/s is marked as a black star and occurs when ${V}_{A}$=2.52 and ${\left|{\mathrm{E}}^{\mathrm{R}}\right|}^{2}$=1056. Any other combinations have shown to reduce the key rate performance and the choices of ${\mathrm{V}}_{\mathrm{A}}$ and ${\mathrm{E}}_{\mathrm{Ref}}^{2}$ should be limited within a certain range to ensure high rates of secure key exchange (*V*_*A*_ ε [2.05, 3.10] and ${\left|{\mathrm{E}}^{\mathrm{R}}\right|}^{2}$ ε [750, 1400]). We also demonstrate the corresponding excess noise ${\upxi }_{\mathrm{e}}$ performance with respect to different ${V}_{A}$ and ${\left|{\mathrm{E}}^{\mathrm{R}}\right|}^{2}$ combinations in Fig. 2b. At the key rate peak represented as a black star, the excess noise ${\upxi }_{\mathrm{e}}$ is 0.083. One can note from the figure that a low ${\upxi }_{\mathrm{e}}$, and hence a low ${\upchi }_{\mathrm{BE}}$, does not necessarily lead to a high key rate since ${\mathrm{V}}_{\mathrm{A}}$ also participates in the mutual information evaluation ${\mathrm{I}}_{\mathrm{AB}}$ between Alice and Bob. For instance, the region of low excess noise operation, ${\upxi }_{\mathrm{e}}$ <${10}^{-3}$, shown as the dark blue region in Fig. 2b, can be achieved when ${\mathrm{V}}_{\mathrm{A}}$ and ${\mathrm{E}}_{\mathrm{Ref}}^{2}$ are both small enough. However a small ${\mathrm{V}}_{\mathrm{A}}$ will significantly reduce ${\mathrm{I}}_{\mathrm{AB}}$, which in turn results in a tiny or even null useful key exchange. Similarly, even with a fixed ${\mathrm{V}}_{\mathrm{A}}$, either a too small or too large ${\left|{\mathrm{E}}^{\mathrm{R}}\right|}^{2}$ will also increase the system excess noise ${\upxi }_{\mathrm{e}}$ and hence lead to poor system performance. The highest achievable key rate for each ${\mathrm{V}}_{\mathrm{A}}$ choice occurs at the optimal ${\left|{\mathrm{E}}^{\mathrm{R}}\right|}^{2}$ is located at the valley line shown in Fig. 2b. As a result, both ${\mathrm{E}}_{\mathrm{ref}}^{2}$ and ${\mathrm{V}}_{\mathrm{A}}$ need calibration, and it is of utmost importance to implement the combined-optimization method to find the global maximum key rate point. In our system, these two parameters can be freely adjusted within the software.

### Combined-optimization method experimental validation

**Figure 3:**
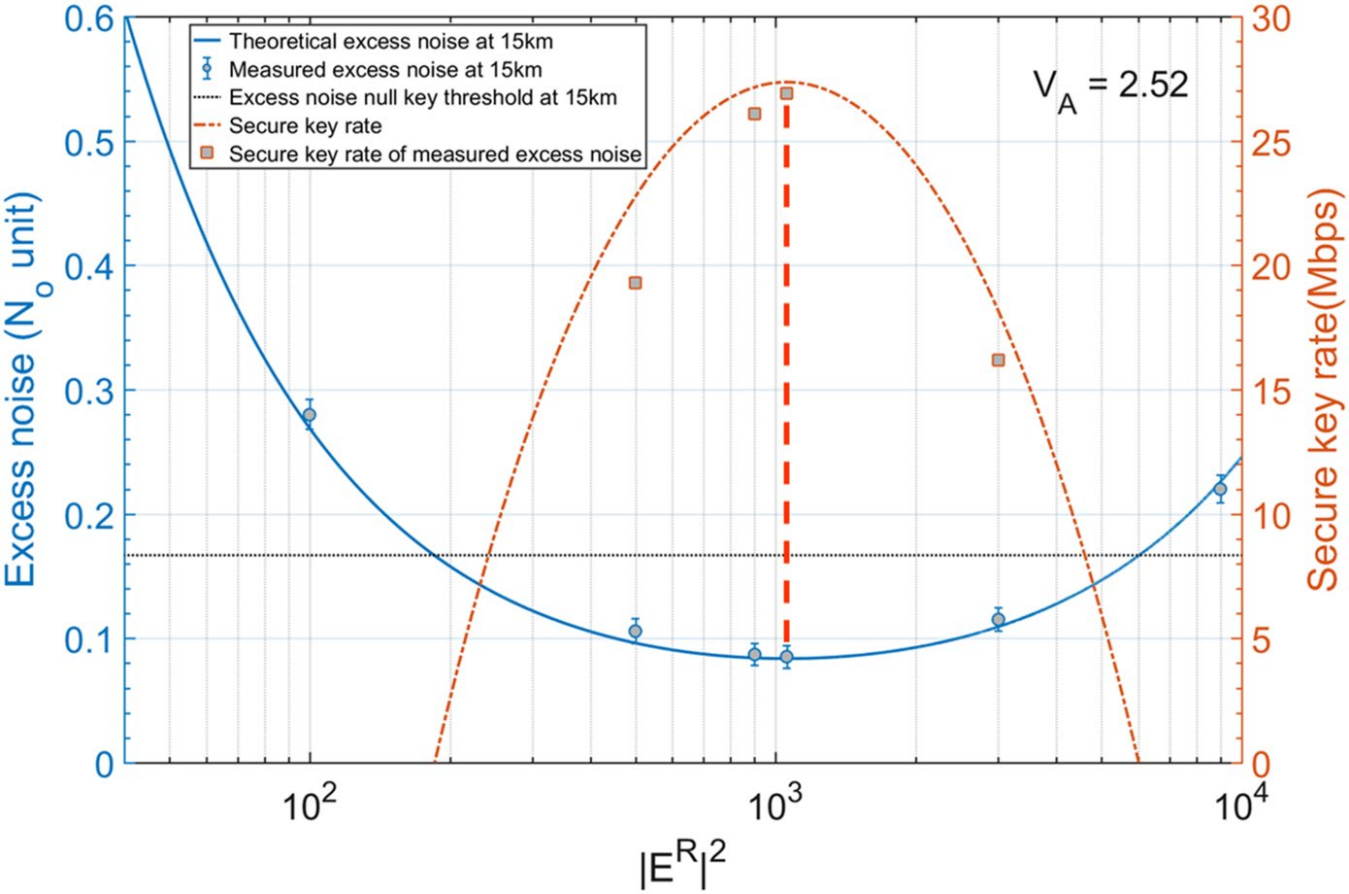
Experimental validation of noise model and optimization method by collecting excess noise at different ${\left|{\mathrm{E}}^{\mathrm{R}}\right|}^{2}$ with a fixed optimal ${V}_{A}=2.52$ and L = 15 km. The blue solid line represents the theoretical excess noise evaluation and the grey circles with ± 0.01 error bars denote the measured excess noises with a data block size of ${10}^{7}$. The dashed orange line represents the theoretical secure key rate performance and the grey squares are the corresponding secure key rate with measured excess noises. The black line is the null key threshold with ${\upxi }_{\mathrm{e}}$=0.167. The peak secure key rate occurs at the minimum excess noise point with ${\left|{\mathrm{E}}^{\mathrm{R}}\right|}^{2}$=1050. Other parameters are listed in Table 1 in the “Methods” section.

In order to demonstrate the effectiveness of the combined-optimization method and verify the relationship between ${\left|{\mathrm{E}}^{\mathrm{R}}\right|}^{2}$ and the excess noise ${\upxi }_{\mathrm{e}}$ at the pre-defined ${\mathrm{V}}_{\mathrm{A}}$, we compare the simulated excess noise ${\upxi }_{\mathrm{e}}$ with experimental measurements for a range of reference pulse intensities over a 15 km optical fibre link at the optimal ${V}_{A}$=2.52. The excess noise measurement is similar to that in conventional CVQKD systems. We evaluated the excess noise using a linear model ${\mathrm{y}}_{\mathrm{i}}=\sqrt{\frac{\mathrm{\eta T}}{2}}{x}_{i}+z$, where ${\mathrm{x}}_{\mathrm{i}}$ and ${\mathrm{y}}_{\mathrm{i}}$ are correlated variables at Alice and Bob, and $\mathrm{z}$ is the overall noise following a zero centred normal distribution with a variance of ${\upsigma }^{2}=\frac{\eta T}{2}{\xi }_{e}+1+{v}_{ele}$. By exchanging 10% of the corrected raw data, we can estimate the overall excess noise ${\xi }_{e}$ of our system. The excess noise results, along with the corresponding calculated key rates, are shown in Fig. 3. As can be seen, the measured excess noise (grey circles) with ± 0.01 error at six different ${\left|{\mathrm{E}}^{\mathrm{R}}\right|}^{2}$ are a good fit to the theoretical excess noise derivation (blue line) calculated by Eq. (16). The minimum excess noise in the experiment is 0.085 at ${\left|{\mathrm{E}}^{\mathrm{R}}\right|}^{2}$=1050. The slightly higher experimental excess noise is likely due to the small underestimation in the original system excess noise. The excess noise exceeds the null key threshold ${\upxi }_{\mathrm{e}}=0.167$ when ${\left|{\mathrm{E}}^{\mathrm{R}}\right|}^{2}$ is either smaller than 185 or larger than 6000, which proves the relationship between ${\left|{\mathrm{E}}^{\mathrm{R}}\right|}^{2}$ and ${\upxi }_{\mathrm{e}}$. The theoretical secure key rate is drawn as an orange dashed line, and the experimental key rates predicted with the measured excess noise values are represented as grey squares. The experimental key rates are slightly smaller than simulation results but overall follow the theoretical trend. The key rate is verified to be inversely proportional to the excess noise at a fixed ${\mathrm{V}}_{\mathrm{A}}$ and the highest key rate of 26.9 Mb/s occurs at the minimum excess noise. Therefore, the practical optimal ${\left|{\mathrm{E}}^{\mathrm{R}}\right|}^{2}$ is confirmed as 1050 at ${\mathrm{V}}_{\mathrm{A}}=2.52$ which validates the feasibility of our proposed combined-optimization method.

### Excess noise stability and secure key rate performance

**Figure 4:**
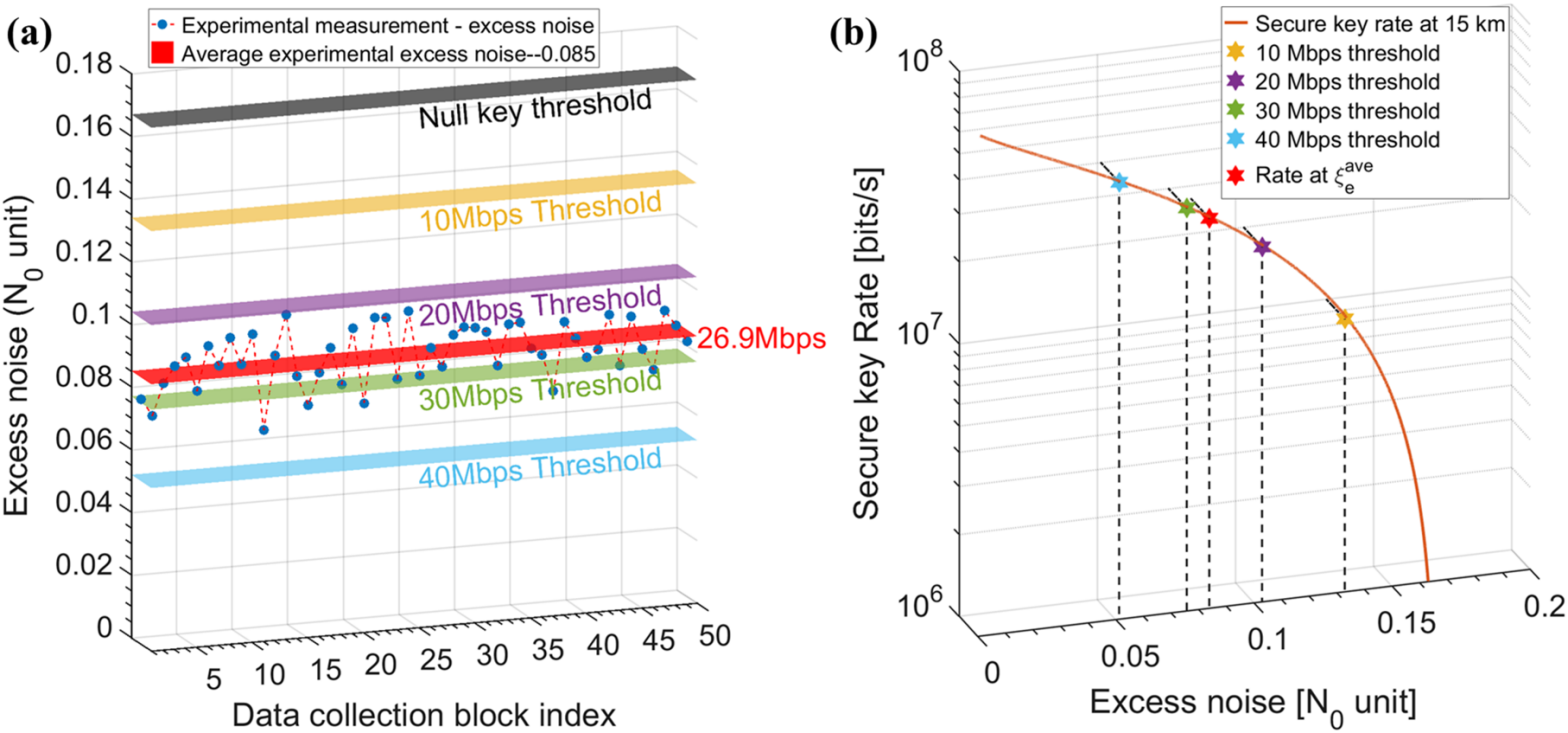
Excess noise stability in our LLO CVQKD system. (**a**) Experimental measurement of excess noise. The blue dots represent the collected excess noise at each data block and the red surface is their average value. Surfaces with different colours are theoretical secure key rate thresholds for null key, 10 Mb/s, 20 Mb/s, 30 Mb/s and 40 Mb/s secret key rates. (**b**) Secure key rate with respect to different excess noises where L = 15 km and ${\mathrm{f}}_{\mathrm{rep}}=500$ MHz. The stars denote the secure key rates at different excess noise thresholds shown in Fig. 4a and the red star represents the secure key rate with an average measured excess noise ${\upxi }_{\mathrm{e}}^{\mathrm{ave}}$=0.085.

With the setup and optimal parameters discussed above, we investigate the practical system stability in terms of fluctuations in real-time excess noise ${\xi }_{e}$. The experimental results of excess noise over 50 consecutive data blocks (each with ${10}^{7}$ signals) over the 15 km SMF optical fibre link are collected. The received data in each data block follows a Gaussian distribution. The phase noise variance is suppressed to ${V}_{phase}\approx 0.01$ by the phase recovery scheme. As shown in Fig. 4a, the excess noise measurements of each data block are marked as blue dots and can be seen to take values between 0.06 to 0.1. A small variation in the real-time excess noise is caused by the output fluctuation of the LO and the statistical errors in the parameter estimation process. The calculated average excess noise ${\upxi }_{\mathrm{e}}^{\mathrm{ave}}$ shown as the red surface is 0.085, which is almost the same as the theoretical excess noise 0.083 obtained by the combined-optimization method. In order to demonstrate the key rate performance of the collected excess noise values intuitively, we draw several secure key rate thresholds in Fig. 4a. These thresholds are obtained from Fig. 4b where the asymptotic secure key rate estimation as a function of ${\xi }_{e}$ is plotted. The null key, 10 Mb/s, 20 Mb/s, 30 Mb/s and 40 Mb/s estimated secret key rates can be achieved by excess noise values of 0.167, 0.134, 0.104, 0.076 and 0.052 respectively. The average measured excess noise 0.085 contributes to 26.9 Mb/s predicted secure key rate and all our measured excess noises are less than 0.1, which guarantee a stable secret key rate generation greater than 20 Mb/s in the asymptotic regime. It is worth noting that the excess noise can be further reduced if complicated multiplexing and separate detection techniques are employed.

**Figure 5:**
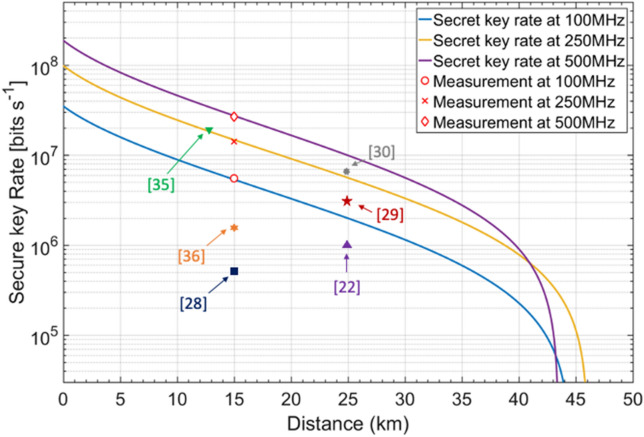
The secret key rate performance of our LLO CVQKD system. The secret key rate as a function of transmission distance at 100 MHz, 250 MHz and 500 MHz repetition rates, together with the average experimental measurements of excess noises over a 15 km SMF fibre. The red circle, cross and diamond represent the estimated key rate of 5.5 Mbps, 14.2 Mbps and 26.9 Mbps at 100 MHz, 250 MHz and 500 MHz repetition rates respectively. The data block size is ${10}^{7}$. ${\mathrm{V}}_{\mathrm{A}}$ and ${\left|{\mathrm{E}}^{\mathrm{R}}\right|}^{2}$ are optimized every 0.1 km using the combined optimization method and other parameters are listed in “Methods”.

The simulation predictions of secret key rate performance at 100 MHz, 250 MHz and 500 MHz repetition rate with respect to different transmission distances, together with measured results from the experiments, are shown in Fig. 5. Previous LLO CVQKD asymptotic regime experimental results are also shown in Fig. 5 for performance comparison. The combined-optimization method is applied every 0.1 km to adjust ${\mathrm{V}}_{\mathrm{A}}$ and ${\left|{\mathrm{E}}^{\mathrm{R}}\right|}^{2}$, which ensures the optimal asymptotic theoretical key rates (solid lines) under each repetition rate scenarios. All other experimental parameters are listed in Table 1 and remain unchanged over different transmission distances. We experimentally measured the excess noise of 50 data blocks over the 15 km link at these three repetition rates. Based on the average excess noise values ${\upxi }_{100\mathrm{M}}^{\mathrm{ave}}=0.084$, ${\upxi }_{250\mathrm{M}}^{\mathrm{ave}}=0.082$ ${\upxi }_{500\mathrm{M}}^{\mathrm{ave}}=0.085$ collected in the experiments, the asymptotic final key rates are computed as 5.5 Mb/s (red circle), 14.2 Mb/s (red cross) and 26.9 Mb/s (red diamond) at 100 MHz, 250 MHz and 500 MHz repetition rate respectively. The experimental results match the** simulation results of the theoretical model, and we can further predict that the transmission distance of our 500 MHz system can reach 43 km beyond which the excess noise will rise above the null key threshold. It can be seen that the final predicted key rate is not perfectly linear with the repetition rate and the excess noise obtained at 250 MHz repetition rate is lower than the other two cases. This is because the phase drift noise ${\upxi }_{\mathrm{drift}}$ plays an important role at relatively low rates and decreases with the increase of repetition rate. However, when the repetition rate goes higher, the effective resolution bit of DAC, and the electronic noise start to limit the key rate. The experimental results have verified the feasibility of implementing an asymptotic 20 + Mbps low-complexity LLO-CVQKD system of over 15 km optical fibre link. When finite-size effects, with a data block size of ${10}^{7}$ and 10% data scarification for parameter estimation, are considered following the calculation described in^28^, the average worst excess noise of our system under the finite size effect is around 0.106 and the predicted key rate is 15.9 Mbps. Further increase in the data block size can improve the key rate performance, achieving the key rate of 20.8 Mbps with data block size of ${10}^{8}$ and key rate of 22.7 Mbps with data block size of ${10}^{10}$. Since the bandwidths of all optical components are in order of GHz, our system remains potential for higher key rate by further pushing the repetition rate beyond current limitations.

## Discussion

We present a low complexity 500 MHz pulse repletion rate LLO-CVQKD system that could enable a record asymptotic key rate of 26.9 Mb/s over a 15 km optical fibre to be predicted from experimental results. In terms of the system setup design, we deploy a low-complexity configuration with real-time software control to realize a cost-effective system with a high degree of adjustment flexibility. A set of wideband components and a continuous wave LO are used to overcome the repetition rate limitation of previous LLO CVQKD systems. The combined-optimization method and the real-time shot noise calibration are integrated through comprehensive noise model analysis to achieve a stable low excess noise performance. Investigation of the post-processing procedures and block size increments will be carried out in the future. Currently, the repetition rate is limited mainly by the bandwidth of the electrical signal generation equipment, and there is no fundamental reason why GHz pulse repetition rate systems are not achievable. This work verifies the feasibility of 20 Mb/s secure key rates in LLO CVQKD and paves the way for the cost-effective quantum communications in practical applications.

## Methods

### Noise model analysis

Optimal performance requires a comprehensive noise analysis, especially for the excess noise that arises from the experimental imperfections and noise introduced by Eve’s manipulation^37,38^. We derive the noise model of our system at high repetition rates and all noise sources presented here are referred to as the channel input noise and are normalized to shot noise unit ${N}_{o}$.

#### Phase noise

In an LLO CVQKD system, owing to the random relative phase drift between two free-running lasers, the main experimental challenge is to agree on a common phase reference between Alice and Bob. According to Eq. (1), even though the phase recovery scheme has been employed for the drift amendment, the phase rotation of the quantum signals cannot be completely corrected through the phase drift estimation of the reference pulse. We have considered the system security where the phase noise is not trusted. The phase noise ${\upxi }_{\mathrm{phase}}$ is one of the most critical contributions to the excess noise that limits the performance of the LLO CVQKD system. Under the GMCS protocol with ${\mathrm{V}}_{\mathrm{A}}$, the phase noise is assumed to be Gaussian distributed with a variance ${\mathrm{V}}_{\mathrm{phase}}$ which represents the variance of residual phase error $\phi$ ^23^. The ${\upxi }_{\mathrm{phase}}$ has contributions from three uncertainties: (1) phase estimation error noise ${\upxi }_{\mathrm{est}}$, (2) drift noise induced from laser sources ${\upxi }_{drift}$, and (3) drift noise induced from the propagation length difference between quantum signals and reference pulses ${{ \xi }}_{{{\text{Ch}}}}$. Their relationship can be modeled as:

6 $$\upxi _{{{\text{phase}}}} = \upxi _{{{\text{est}}}} + \upxi _{drift} + \upxi _{{{\text{Ch}}}} \approx V_{A} *V_{phase}$$

where $${\text{V}}_{{{\text{phase}}}}$$ is:

7 $${\text{V}}_{{{\text{phase}}}} = var\left( {\varphi_{est}^{R} - {\varphi }^{S} } \right) = var\left( \phi \right)$$

The phase estimation error noise ${\upxi }_{{{\text{est}}}}$ is originated from reference pulse quantum uncertainty ${\text{V}}_{{{\text{est}}}}$ at the detector due to the fundamental shot noise and total noise ${\upchi }_{{{\text{tot}}}}$ of our system. It describes noise induced from the deviation between the exact phase ${{\varphi }}^{{\text{R}}}$ and the measured phase ${{\varphi }}_{{{\text{est}}}}^{{\text{R}}}$ of the reference pulse. The value is inversely proportional to the reference pulse intensity $\left| {{\text{E}}^{{\text{R}}} } \right|^{2}$ at the Bob output^24^ as,

8 $$\upxi _{{{\text{esti}}}} = {\text{V}}_{{\text{A}}} *var\left( {{\varphi }_{{{\text{est}}}}^{{\text{R}}} - {\varphi }^{{\text{R}}} } \right) = \frac{{V_{A} *\left( {\chi_{tot} + 1} \right)}}{{\left| {E^{R} } \right|^{2} }}$$

The drift noise ${\upxi }_{drift}$ results from the phase frames instability of two independent free-running laser sources with the spectrum linewidths ${\Delta v}_{{\text{A}}}$ and ${{ \Delta v}}_{{\text{B}}}$ at Alice and Bob respectively. Due to the temporal separation of the generation of the quantum signal and reference pulse, the relative phase drift ${\upxi }_{drift}$ can be modeled as a Gaussian stochastic process centreed at the laser central frequency. The value can be characterized by the variance of phase drift ${\text{V}}_{{{\text{drift}}}}$ within a half repetition interval $\frac{{{\text{T}}_{{{\text{rep}}}} }}{2}$:

9 $$\upxi _{{{\text{drift}}}} = {\text{V}}_{{\text{A}}} * var\left( {\uptheta _{A}^{{\text{R}}} - \theta_{A}^{S} } \right) = {\text{V}}_{{\text{A}}} *\uppi \frac{{\Delta v_{A} + \Delta v_{B} }}{{f_{rep} }}$$

In our system, with a narrow laser linewidth of 100 kHz and a high repetition rate $f_{rep}$ = 500 MHz, the ${\text{V}}_{{{\text{drift}}}}$ is suppressed within the order of $10^{ - 3} \,{\text{rad}}^{2}$.

The ${\upxi }_{{{\text{Ch}}}}$ represents the relative phase drift accumulated by the quantum signal and reference pulse during propagation. The value is assumed to be dominated by the optical path length difference of two components. In our system, the optical path of two components are the same, the phase rotations accumulated by two components ${\uptheta }_{{{\text{Ch}}}}^{{\text{S}}}$ and ${\uptheta }_{{{\text{Ch}}}}^{R}$ are hence identical.

10 $$\xi_{Ch} = {\text{V}}_{{\text{A}}} *var\left( {\uptheta _{{{\text{Ch}}}}^{R} -\uptheta _{{{\text{Ch}}}}^{{\text{S}}} } \right) \approx 0$$

#### Conversion noise

In our system, two types of conversion noise are induced by the imperfect conversion between digital bits and analogue voltages. The first occurs at Alice’s modulation voltage preparation. When the AWG and wideband drivers are used to translate signal bits into analogue modulation voltages, the modulation voltage fluctuation ${{ \Delta U}}_{{{\text{mod}}}}$ is caused by the imperfect conversion of those components and the adjacent sample interferences in the amplification process. Based on the analysis in^27^, such fluctuation noise ${{ \xi }}_{{{\text{fluc}}}}$ can be evaluated as:

11 $$\xi_{fluc} = V_{A} \left( {\pi \frac{{\Delta {\text{U}}_{{{\text{mod}}}} }}{{U_{\pi } }} + \frac{1}{2}\pi^{2} \frac{{\Delta {\text{U}}_{{{\text{mod}}}}^{2} }}{{U_{\pi }^{2} }}} \right)^{2}$$

where ${\text{U}}_{{\uppi }}$ is the voltage assigned to the phase modulator to achieve a ${\uppi }$ phase modulation.

The second conversion noise is the ADC quantization noise $\xi_{ADC}$ that appears in Bob’s detection process. The output voltages of BHDs are sent to an oscilloscope and digitized by the ADCs. Also, with the same sampling rate, the effective resolution bit number ${\text{n}}_{{{\text{eff}}}}$ of ADC decreases with the increment of the ADC input frequency, specifically 1 bit of ${\text{n}}_{{{\text{eff}}}}$ is lost for every doubling of the ADC input frequency^39^. Such $\xi_{ADC}$ can be evaluated as^27,40^

12 $$\xi_{ADC} = \frac{{ut_{s} }}{{hfg^{2} \rho^{2} P_{LO} \eta T}}*\frac{{U_{full}^{2} }}{{12 \times 2^{{2n_{eff} }} }}$$

where u = 1 for homodyne detection and u = 2 for heterodyne detection, $t_{s}$ is the signal pulse duration, $U_{full}$ is the full voltage range of the ADC, $h$ is Planck’s constant. $f$ is the laser frequency, $g$ is the gain factor of the transimpedance amplifier (TIA, V/A), $\rho$ is the photodiode responsivity (A/W), $P_{LO}$ is the LO power at Bob, and ${\text{n}}_{{{\text{eff}}}}$ is the effective number of bit of the ADC at our system pulse rate. To recover all quantum states correctly, $U_{full}$ needs to cover the full voltage range of the received signals, which means $U_{full}$ needs to be larger than the reference pulse voltage range $U_{R}$:

13 $$U_{full} \ge U_{R} = \sqrt {g\rho *\frac{{\left| {E^{R} } \right|^{2} hf}}{{t_{s} }}*\eta T} *\sqrt {g\rho P_{LO} }$$

Combine Eq. (12) and Eq. (13), the lower bound of $\xi_{ADC}$ can be calculated by:

14 $$\xi_{ADC}^{min} = \frac{{u\left| {E^{R} } \right|^{2} }}{{12*2^{{2n_{eff} }} }}$$

As can be seen for CVQKD systems operating at high repetition rates, due to the reduction in $n_{eff}$ at high speed, the ADC quantization noise will play an essential role in determining the excess noise and hence the system performance.

#### AM dynamic range noise

During the signal preparation stage at Alice, the interleaved intense reference pulses and the weak quantum signals are generated using a single amplitude modulator (AM2) with a finite dynamic range. After we generate the reference pulse, the finite dynamic range of AM2 will induce amplitude leakage on the weak quantum signal and introduce AM dynamic range noise $\xi_{d}$ on the quadratures of quantum signals^23^. With a reference pulse intensity $\left| {{\text{E}}^{{\text{R}}} } \right|^{2}$ and an extinction ratio ${\text{r}}_{{\text{e}}}^{AM2}$(in dB), the ${\upxi }_{{{\text{AM}}}} { }$ can be approximated as

15 $$\xi_{d} = \left| {{\text{E}}^{{\text{R}}} } \right|^{2} 10^{{{ - r_{e}{{\text{AM2}}}} /10}}$$

Compared with the large photon-leakage noise induced by the split and combination of quantum signal and reference pulse in the delay-line scheme^28^, our system does not require split and combination interferometer and delay line at both Alice and Bob and hence has zero photon-leakage noise. Other noise contributions such as relative intensity noise, common-mode rejection ratio noise and BHD imbalanced drift noise, are deemed small enough in our experimental setup to be included within the original system excess noise $\xi_{ori}$ = 0.01. The overall excess noise model can be described as:

16 $$\xi_{e} = \xi_{ori} + \xi_{esti} + \xi_{drift} + \xi_{ch} + \xi_{ADC} + \xi_{fluc} + \xi_{d }$$

#### Electronic noise

In this paper, we adopt the assumption of the trusted detector model widely used in an LLO CVQKD system^20–26^, where the electronic noise ${\mathrm{V}}_{\mathrm{ele}}$ and the efficiency associated with the detector $\upeta$ are well-protected from Eve. The electronic noise arises from the thermal noise inside the BHD. The electronic to shot noise ratio should be low enough to achieve a precise quantum signal detection. The electronic noise in shot noise unit at the inputs of detectors can be expressed as^27,40^:

17 $$V_{ele} = \frac{{NEP_{ele}^{2} B}}{{r_{LO} hfP_{LO} }}$$

where B is the bandwidth of the detector, which is chosen to be $3{\text{f}}_{{{\text{rep}}}}$ in our system, ${\text{NEP}}_{{{\text{ele}}}}$ is the noise equivalent power of the BHD at B, and $r_{LO}$ is the rate of incoming signals interfering with LO, which is equivalent to pulse generation rate in this system. The electronic noise increases with system repetition rate, but it can be improved with an increased LO power. The electronic noise of our system is verified experimentally as 0.1.

The linear model to represent the quadrature values measured by Bob with a transmittance ${\text{T }}$ and a heterodyne detection efficiency ${\upeta }$ is derived as:

18 $$\left( {\begin{array}{*{20}c} {X_{B}^{S} } \\ {P_{B}^{S} } \\ \end{array} } \right) = \sqrt {\frac{T\eta }{2}} \left[ {\left( {\begin{array}{*{20}c} {X_{\xi e} + X_{N} } \\ {P_{\xi e} + P_{N} } \\ \end{array} } \right) + \left( {\begin{array}{*{20}c} {\cos (\varphi_{est}^{R} ) \sin (\varphi_{est}^{R} ) } \\ { - \sin (\varphi_{est}^{R} ) \cos (\varphi_{est}^{R} )} \\ \end{array} } \right)\left( {\begin{array}{*{20}c} {X_{A}^{S} } \\ {P_{A}^{S} } \\ \end{array} } \right)} \right] + \left( {\begin{array}{*{20}c} {X_{ele} } \\ {P_{ele} } \\ \end{array} } \right)$$

where the rotation matrix applied to the initial signal quadratures accounts for the phase noise while $X_{\xi e}$ and $P_{\xi e}$ represent the phase-noise-exclusive excess noise. $X_{N}$ and $P_{N}$ are vacuum quadratures of unit shot noise variance ${\text{ N}}_{{\text{o}}}$. ${\text{X}}_{{{\text{ele}}}}$ and ${\text{P}}_{{{\text{ele}}}}$ are electronic noise quadratures with variance ${\text{ V}}_{{{\text{ele}}}}$.

All parameters used in our simulation and experiment are listed in Table 1.

**Table 1:** Parameters used in the experiment.

Parameter	Symbol	Value
Detector efficiency	$\upeta$	0.49
Reconciliation efficiency	$\upbeta$	0.95
Pulse duration	$t_{s}$	0.2 ns
Repetition rate	${\text{f}}_{{{\text{rep}}}}$	500 MHz
Pulse rate	${\text{f}}_{{{\text{pulse}}}}$	1 GHz
Effective resolution bit number	${\text{n}}_{{{\text{eff}}}}$	8.6 bits
Voltage for $\pi$ modulation	${\text{U}}_{\uppi }$	4.9 V_pp_
Noise equivalent power	${\text{NEP}}_{{{\text{ele}}}}$	$14\, {\text{pw}}/\sqrt {{\text{Hz}}}$
Detector bandwidth	B	$1.5\,{\text{GHz}}$
Gain factor of TIA	*g*	16 k V/*A*
Photodiode responsivity	$\rho$	$0.9\,{\text{A/W}}$
Modulation voltage fluctuation	$\Delta {\text{U}}_{{{\text{mod}}}}$	$0.15\,{\text{V}}$
Channel length	L	15 km
Attenuation coefficient	$\upalpha _{{\text{n}}}$	0.2 dB/km
AM dynamic range	47	dB
Rate of LO interference	$r_{LO}$	1 GHz
Local oscillator power	$P_{LO}$	$13 \,{\text{dBm}}$
Electronic noise	${\text{V}}_{{{\text{ele}}}}$	$0.1\,{\text{N}}_{{\text{o}}}$
Laser linewidth	$\Delta {v}$	100 kHz

To clearly show the order of magnitude of the different excess noise sources at 500 MHz repetition rate, the contributions of all excess noise sources and their values are listed in Table 2.

**Table 2:** Magnitudes and contributions of the different excess noise sources at 500 MHz repetition rate.

Noise source	Noise magnitude (shot noise unit)	Excess noise contribution (%)
Phase estimation error noise ${\upxi }_{\mathrm{est}}$	0.022	26.5
Drift noise ${\upxi }_{drift}$	0.006	7.2
Channel noise ${\upxi }_{\mathrm{Ch}}$	0	0
Fluctuation noise ${\upxi }_{\mathrm{fluc}}$	0.024	28.9
ADC quantization noise ${\xi }_{ADC}$	0.001	1.3
AM dynamic range noise ${\xi }_{d}$	0.020	24.1
Original system excess noise ${\xi }_{ori}$	0.01	12.0
Overall excess noise	0.083	100

### Secret key rate evaluation

The asymptotic secret key estimation of our LLO-CVQKD scheme follows the same procedures used in a conventional CVQKD system^12^. Under a collective attack with heterodyne detection and reverse reconciliation, the secure key rate ${\text{K}}_{{{\text{col}}}}$ is:

19 $$K_{col} = f_{rep} \beta I_{AB} - \chi_{BE}$$

where ${\upbeta }$ is the reconciliation efficiency, ${\text{I}}_{{{\text{AB}}}}$ is the mutual information between Alice and Bob, and ${{ \chi }}_{{{\text{BE}}}}$ is the upper bound of Eve’s information related to the Holevo bound^41^. $I_{AB}$ with heterodyne detection can be derived through Shannon equation as^42^:

20 $${\text{I}}_{{{\text{AB}}}} = \log_{2} \frac{{V_{B} }}{{V_{B\left| A \right.} }} = \log_{2} \frac{{V_{A} + 1 + \chi_{tot} }}{{1 + \chi_{tot} }}$$

The total noise ${\upchi }_{{{\text{tot}}}}$ of our system has two contributions: the line noise ${\upchi }_{{{\text{line}}}}$, and the imperfect detection noise ${\upchi }_{{{\text{het}}}}$. The line noise ${\upchi }_{{{\text{line}}}}$ induced in the channel consists of transmission loss and all untrusted noises referred to the channel input and can be expressed as ${ }\chi_{line} = \frac{1}{T} - 1 + \xi_{e}$. According to the heterodyne detection setup for reference pulses and quantum signals, the noise introduced by the detector can be expressed as:$\chi_{het} = \frac{{\left[ {1 + \left( {1 - \eta } \right) + 2V_{ele} } \right]}}{\eta }$. The information accessed by Eve ${\upchi }_{{{\text{BE}}}}$ can be evaluated by the Von Neumann entropy S(.)^43^ using the symplectic eigenvalues^44^ of the covariance matrix. The derivation of ${\upchi }_{{{\text{BE}}}}$ in our system follows exactly the same as that of a conventional CV-QKD system, but with an updated excess noise model in $\chi_{line}$. The mutual information between Eve and Bob is given by:

21 $$\chi_{BE} = \mathop \sum \limits_{i = 1}^{2} G\left( {\frac{{\lambda_{i} - 1}}{2}} \right) + \mathop \sum \limits_{i = 3}^{5} G\left( {\frac{{\lambda_{i} - 1}}{2}} \right)$$

where ${\text{G}}\left( {\text{x}} \right) = \left( {{\text{x}} + 1} \right)\log_{2} \left( {{\text{x}} + 1} \right) - {\text{xlog}}_{2} { },$ and ${\uplambda }_{{\text{i}}}$ are the symplectic eigenvalues. Specifically ${\uplambda }_{1,2} = \sqrt {\frac{1}{2}\left( {A \pm \sqrt {A^{2} - 4B} } \right){ }}$ where

22 $$A = V^{2} \left( {1 - 2T} \right) + 2T + T^{2} \left( {V + \chi_{line} } \right)^{2}$$

23 $${\text{B}} = \left[ {{\text{T}}\left( {{\text{V}}\upchi _{{{\text{line}}}} + 1} \right)} \right]^{2}$$

$${\uplambda }_{3,4} = \sqrt {\frac{1}{2}\left( {C \pm \sqrt {C^{2} - 4D} } \right)}$$ and $${\uplambda }_{5} = 1$$, where

24 $${\text{C}} = \frac{1}{{\left( {T\left( {V + \chi_{t} } \right)} \right)^{2} }}\left[ {A\chi_{het}^{2} + B + 1 + 2\chi_{het} \left( {V\sqrt B + T\left( {V + \chi_{line} } \right)} \right) + 2T\left( {V^{2} - 1} \right)} \right]$$

25 $${\text{D}} = \left( {\frac{{{\text{V}} + \sqrt {\text{B}} \chi_{het} }}{{T\left( {V + \chi_{t} } \right)}}} \right)^{2}$$

## Data Availability

The data that support the findings of this study are available in Apollo, the University of Cambridge Repository with the identifier 10.17863/CAM.68384.
